# Synthesis, Structure, Electrochemistry, and Cytotoxic Properties of Ferrocenyl Ester Derivatives

**DOI:** 10.1155/2009/420784

**Published:** 2009-03-24

**Authors:** Li Ming Gao, Ramón Hernández, Jaime Matta, Enrique Meléndez

**Affiliations:** ^1^Department of Chemistry, University of Puerto Rico, P.O. Box 9019, Mayagüez, PR 00681, USA; ^2^Department of Pharmacology, Toxicology and Physiology, Ponce School of Medicine, P.O. Box 7004, Ponce, PR 00732, USA

## Abstract

A series of ferrocenyl ester complexes, varying the lipophilic character of the pendant groups, was prepared and characterized by spectroscopic and analytical methods. The syntheses of Fe(C_5_H_4_CO_2_CH_3_)_2_, Fe(CpCOOCH_3_) (CpCOO CH_2_CH_3_), and Fe(CpCOOCH_2_CH_3_)_2_ are reported. The solid-state structure of Fe(C_5_H_4_CO_2_CH_3_)_2_ has been determined by X-ray crystallography. Fe(C_5_H_4_CO_2_CH_3_)_2_ has the cyclopentadienyl rings virtually in an eclipsed conformation with the pendant groups not completely opposite to each other. Cyclic voltammetry characterization showed that the functionalized ferrocenes oxidize at potentials, E_pa_, higher than ferrocene as a result of the electro withdrawing effect of the pendant groups on the cyclopentadienyl ligand. The cytotoxicities of Fe(C_5_H_4_CO_2_CH_2_CH_2_OH)_2_, Fe(C_5_H_4_CO_2_CH_2_CH=CH_2_)_2_, Fe(C_5_H_4_CO_2_CH_3_)_2_, Fe(CpCOOCH_3_)(CpCOOCH_2_CH_3_), and Fe(CpCOOCH_2_CH_3_)_2_ in colon cancer HT-29 and breast cancer MCF-7 cell lines were measured by the MTT biological viability assay and compared to ferrocene and ferrocenium. Fe(C_5_H_4_CO_2_CH_2_CH=CH_2_)_2_ showed the best IC_50_ values, 180(10) *μ*M for HT-29 and 190(30) *μ*M for MCF-7 cell lines, with cytotoxicities similar to ferrocenium. The cytotoxic data suggest that as we increase the lipophilic character of the functionalized ferrocene, the cytotoxicity improves approaching to the cytotoxic activity of ferrocenium.

## 1. Introduction

Modern organometallic chemistry has been greatly influenced by the accidental discovery of bis(cyclopentadienyl)iron(II) (ferrocene) in 1951 [1, 2]. In fact, ferrocene (Cp_2_Fe) has been the most extensively studied organometallic species. Its applications in catalysis, organic synthesis, and industrial processes are numerous [3–6]. The thermal stability, inert character in concentrated acid and base solutions as well as the redox properties of ferrocene makes this species a versatile compound in many research areas.

Until 1979, the biological properties of metallocenes were unexplored. The report of titanocene dichloride (Cp_2_TiCl_2_) as the first metallocene to possess antitumor activity opened a new area of research, bioorganometallics, which has been developing rapidly in the last twenty eight years [7, 8].

In 1984, Köpf-Maier et al. reported the anticancer activity of ferrocenium complex in Ehrlich ascites tumor [9]. Since then, ferrocene has been studied for potential biological and medicinal applications [8, 10, 11]. Originally, the oxidized species of ferrocene, ferrocenium (Cp_2_Fe^+^) was reported to be responsible for the cytotoxic properties on DNA mediated through its capacity to generate oxygen-free radical species [12, 13]. Ferrocene has been recently reported to have antitumor properties due to the metabolic formation of ferrocenium ions which induces oxidative damage to DNA [14, 15]. As a result, many functionalized ferrocenes have been prepared and tested in cancer cells [11].

**Scheme 1:**
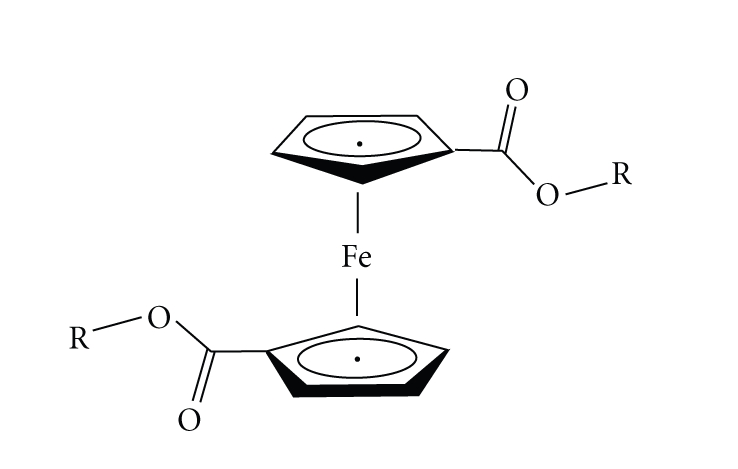
Structure of ferrocenyl ester complexes. R = CH_3_, CH_2_CH_3_, CH_2_CH_2_OH, and CH_2_CH=CH_2_.

We have initiated a project on functionalized ferrocene chemistry, with pendant groups (functional groups, R) on the Cp rings varying their polar and lipophilic characters, see Scheme 1. The objective of this study is to understand how the polar and lipophilic characters of the pendant groups change the anticancer activity of the corresponding ferrocenyl derivatives. We have investigated the synthesis, structure, electrochemistry, and cytotoxic properties of five ferrocenyl esters in HT-29 colon cancer and MCF-7 breast cancer cell lines. The objective of this study is to report these novel findings.

## 2. Results and Discussion

### 2.1. Synthesis and Structure

The syntheses of Fe(C_5_H_4_CO_2_CH_3_)_2_, Fe(C_5_H_4_CO_2_CH_2_CH=CH_2_)_2_, and Fe(C_5_H_4_CO_2_CH_2_CH_2_OH)_2_ have been reported previously [16, 17]. We applied the methodology developed by Busetto et al. to synthesize Fe(C_5_H_4_CO_2_CH_2_CH_3_)_2_ and Fe(C_5_H_4_CO_2_CH_2_CH_3_)(C_5_H_4_CO_2_CH_3_) (mixed ferrocene) [17]. Interestingly, using this synthetic methodology, the reaction of FeCl_2_ and one equivalent of NaC_5_H_4_CO_2_CH_2_CH_3_ and NaC_5_H_4_CO_2_CH_3_, even at room temperature, allowed the selective incorporation of two distinct functionalized Cp ligands, affording the mixed ferrocene complex in good yield without the formation of Fe(C_5_H_4_CO_2_CH_3_)_2_ and Fe(C_5_H_4_CO_2_CH_2_CH_3_)_2_.

**Scheme 2:**
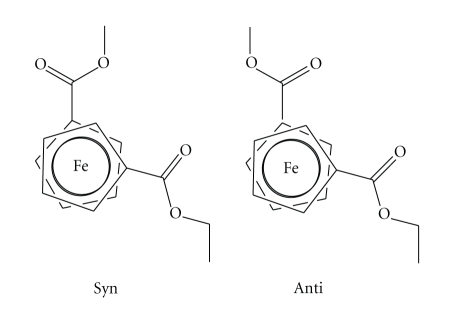
Conformational diastereoisomers of Fe(C_5_H_4_CO_2_CH_2_CH_3_)(C_5_H_4_CO_2_CH_3_).

The NMR, IR, and elemental analysis corroborate the identities of the ferrocenyl ester complexes. The IR spectral data showed *ν*(C=O) broadbands at 1708 cm^−1^ (Fe(C_5_H_4_CO_2_CH_2_CH_3_)_2_ and 1712 cm^−1^ (Fe(C_5_H_4_CO_2_CH_2_CH_3_)(C_5_H_4_CO_2_CH_3_) corresponding to the carbonyl groups of the esters. In the ^1^H NMR spectrum, Fe(C_5_H_4_CO_2_CH_2_CH_3_)_2_ exhibits two set of resonances at 4.42 and 4.84 ppm. These Cp protons exhibit coupling belonging to an AA′BB′ spin system. For the Fe(C_5_H_4_CO_2_CH_2_CH_3_)(C_5_H_4_CO_2_CH_3_), the ^1^H NMR spectrum shows two sets of resonances at 4.42 and 4.85 ppm corresponding to the Cp protons of C_5_H_4_CO_2_CH_2_CH_3_ and C_5_H_4_CO_2_CH_3_ rings. While we might expect four sets of resonances in the vinyl region corresponding to two different substituted Cp rings, the fact that we observed only two sets suggests overlapping between these Cp proton signals. Particularly interesting in the ^13^C NMR spectrum, at room temperature, two sets of resonances for each carbon atom are observed in a ratio of 1:1. This suggests that two possible conformational diastereoisomers, *syn and anti*, coexist in solution as discussed below (Scheme 2) [18].

In solution, rapid ring oscillation about Cp–Fe–Cp axis allows H_2_ and H_5_ (H_2_′ and H_5_′) and H_3_ and H_4_ (H_3_′ and H_4_′) to become equivalents. This situation applies for both substituted Cp rings. Since both Cp rings have ester groups, overlapping of the H_2_/H_5_ and H_2_′/H_5_′, H_3_/H_4_ and H_3_′/H_4_′ signals in the ^1^H NMR spectrum is expected. However, the position of the ester groups yields to conformational diastereoisomers, *syn* and *anti*, which differ in the orientation of the carbonyl groups [18]. This condition applies when the C(Cp)–C(CO) bond is slow in the NMR scale time. When the rotation is fast, having enough energy to overcome the rotational barrier of the C(Cp)–C(CO) bond, such stereolabile chirality disappears. In the ^13^C NMR spectrum, the carbonyl orientation (*syn* and *anti*) makes significantly different magnetic environment on the Cp carbons as well as on the carbonyl groups such that two sets of resonances corresponding to *syn* and *anti* conformers can be observed. Variable temperature NMR studies, in toluene-d_8_, showed that at 80°C in the ^1^H spectrum, the multiplet signal at 4.85 ppm became a single line and the multiplet at 4.43 ppm broadened. These signals belong to the Cp rings. In the ^13^C NMR spectrum at 80°C, the carbon signals of one conformer begin to increase in intensity at expense of the other conformer, reaching a ratio of 1:2. This suggests that conformational diastereoisomers, *syn* and *anti*, at room temperature, have very similar stability but at high temperature, one diastereoisomer (presumably) *anti* becomes more populated.

In general, the present synthetic route developed by Busetto et al. [17] for the ferrocenyl ester complexes (Cp functionalization and formation of the corresponding functionalized ferrocene) represents a convenient alternative procedure for the functionalization of metallocenes and has been used by our group to synthesize functionalized titanocenes [19].

**Figure 1:**
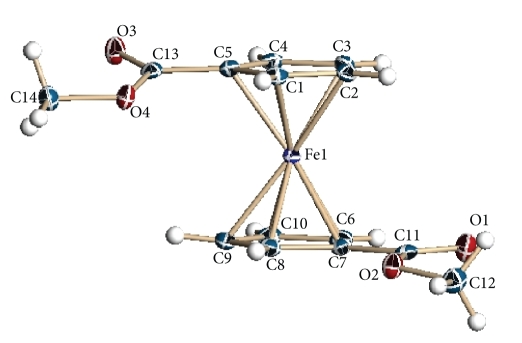
Solid-state structure of Fe(C_5_H_4_CO_2_CH_3_)_2_ drawn 50% thermal ellipsoids. Average Fe-Cp bonds, Fe(C_1_–C_5_) 2.049(28) Å and Fe(C_6_–C_10_) bond 2.048 (11) Å. Torsion angles for C(8)–C(7)–C(11)–O(2) 6.9(4)° and O(4)–C(13)–C(5)–C(1) 15.2(4)°.

The solid-state structure of Fe(C_5_H_4_CO_2_CH_3_)_2_ was investigated by single-crystal X-ray diffraction, see Figure 1. Crystal data and structure refinement are summarized in the Supplementary Material available online at doi:10.1155/2009/420784 (Cambridge Crystallographic Data Centre and the deposition number is CCDC 705387). Bonding parameters are included in Supplementary Table 1.

As shown by X-ray, this ferrocene is a sandwich complex with the cyclopentadienyl ligands adopting an eclipsed conformation and with the pendant groups not completely opposite to each other. The average Fe–C(Cp) distances for Fe(C_5_H_4_CO_2_CH_3_)_2_ are 2.049 and 2.048 $\overset{\prime }{Å}$ which is very similar to those reported for ferrocene, 2.045 $\overset{\prime }{Å}$ [20]. The shortest Fe–C(Cp) bond distances are on C(5) and C(7). The shorter bond distances could be attributed to the inductive (electro-withdrawing) effect of the ester groups on these carbon atoms. The ester groups are not coplanar with the Cp ring, one of the OCH_3_ is bent toward the iron atom (below the Cp plane) with a torsion angle of 15.2° while the other is bent away from the iron atom by 6.9°. The C(Cp)–C(CO) bonds (C(5)–C(13) and C(7)–C(11)) are somewhat shorter than a typical C-C single bond, 1.477(4) versus 1.54 $\overset{\prime }{Å}$ (for a single bond), suggesting these bonds have partial double bond character as a result of conjugation with the Cp *π* system [21]. The later might explain the high C(Cp)–C(CO) rotational barrier encountered in the Fe(C_5_H_4_CO_2_CH_2_CH_3_)(C_5_H_4_CO_2_CH_3_) complex. In addition, the two CO_2_Me groups are *anti* to each other.

While the solid structure of Fe(C_5_H_4_CO_2_CH_3_)_2_ has been reported previously, our solid-state structure has a fundamental difference to that published by Cetina et al. [22]. That is, in the lattice, they determined a hydrogen bonding network between the carbonyl group and the CH_3_ of the adjacent molecule. We could not determine such hydrogen bonding network between the carbonyl group and the CH_3_ of the adjacent molecule even though our data was collected at lower temperature (173 K versus 293 K) and we obtained better refinement parameter (R (I > 2 sigma (I)) 0.0397 versus 0.046).

### 2.2. Cyclic Voltammetry

**Figure 2:**
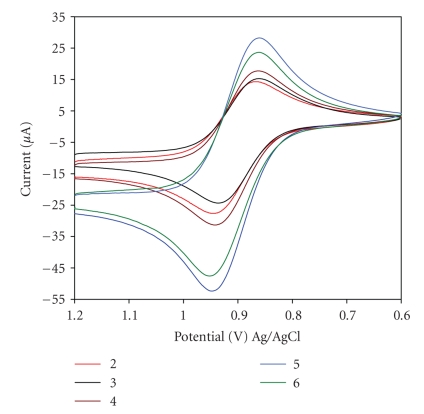
Cyclic voltammograms of complexes 2: Fe(CpCOOMe)_2_, 3: Fe(CpCOOEt)_2_, 4: Fe(CpCOOMe)(CpCOOEt), 5: Fe(CpCOOCH_2_CH=CH_2_)_2_, and 6: Fe(CpCOOCH_2_CH_2_OH)_2_ (2 × 10^−2^ M) in CH_3_CN (1 × 10^−3^ M B_u4_NPF_6_) at room temperature. The working electrode was a platinum disk, reference electrode was Ag/AgCl, and the scan rate was 100 mv/s.

Electrochemical characterization of the subject complexes was performed by mean of cyclic voltammetry (CV). It is well known that ferrocene easily undergoes one electron oxidation to form ferrocenium (Cp_2_Fe^+^) in a reversible manner. Thus, we investigated the functionalized ferrocenes electrochemical behaviors and compared to ferrocene in organic solvent. Table 1 presents the CV results. As it can be seen in the cyclic voltammograms (Figure 2), ferrocene and Fe(C_5_H_4_CO_2_CH_3_)_2_, as well as all the remaining functionalized ferrocenes undergo a reversible redox process with an *i*_*pa*/_ *i*_*pc*_ ratio close to one. All the functionalized ferrocene demonstrated oxidation potentials, *E*_*pa*_, higher than ferrocene. This is the result of the electro-withdrawing (inductive) effect of the ester groups on the Cp rings. Since ferrocene is known to undergo one-electron redox process, it can be deduced that the subject complexes also undergo one-electron redox change. Thus, this major redox process on the functionalized ferrocenes is associated to the ferrocene/ferrocenium couple.

### 2.3. Cytotoxic Studies—3-(4,5-dimethylthiazol-2-yl)-2,5-diphenyltetrazolium Bromide (MTT): Colorimetric Assay for Cytotoxicity Analysis on the Colon Cancer HT-29 and Breast MCF-7 Cell Lines

**Figure 3:**
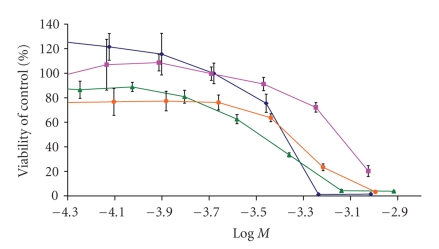
Dose-response curves for functionalized cyclopentadienyl ferrocenes against HT-29 cells at 72 hours drug exposure. Legend: ferrocene-diamonds, bis(carbomethoxy)-squares, bis(carboethoxy)-triangles, and (carbomethoxy)(carboethoxy)-circles.

The cytotoxicities of the ferrocenyl ester complexes on the HT-29 colon cancer and MCF-7 breast cancer cell lines were measured using a slightly modified MTT assay [23, 24]. Ferrocene was initially evaluated at time intervals of 72, 96, and 120 hours at concentrations that ranged from 10–1200 *μ*M, in HT-29, to determine its optimal activity (Supplementary Figure 1S). Ferrocene displayed comparable activity at all three time intervals, with an IC_50_ value of 3.6 × 10^−4^ M. Since exposing the cells to ferrocene at longer periods of time did not increase its cytotoxic activity, all subsequent experiments were performed at a drug exposure time of 72 hours. Ferrocene, ferrocenium, and the functionalized complexes (–CO_2_R, R=Me, Et) were tested in concentrations which ranged from 10–1200 *μ*M. In addition, two control experiments were run 100% Medium and 5% DMSO/95% Medium. Both control experiments behaved identical demonstrating that 5% DMSO in the Medium does not have any cytotoxic effect on these cells. Table 2 summarizes the results of the cytotoxicity experiments and Figure 3 depicts the cytotoxic curves from MTT assays showing the effect of the ferrocene complexes on the viability of HT-29 colon cancer cell line. The IC_50_ value represents the concentration of the ferrocene at which the cell growth is inhibited by 50%. It can be noted that bis(carboethoxycyclopentadienyl)ferrocene showed activity comparable to ferrocene, with values of 370 and 360 *μ*M, respectively but it has less cytotoxicity than ferrocenium (180 *μ*M). In contrast, (carboethoxycyclopentadienyl)-(carbomethoxycyclopentadienyl)-ferrocene and the bis(carbomethoxycyclopentadienyl)-ferrocene exhibited less cytotoxic activity than ferrocene and ferrocenium. The carbomethoxy functionalization has been shown previously to inactivate or lower the cytotoxic activity of resulting titanocene complexes [19]. The present results clearly show how the presence of the carbomethoxy substituent in the cyclopentadienyl ring lowers the activity of ferrocene in a stepwise manner, being the bis(carbomethoxy)ferrocene less active than (carbomethoxy)(carboethoxy)ferrocene. The carboethoxy functionalization does not improve the activity of the ferrocene complexes as compared to ferrocene.

**Figure 4:**
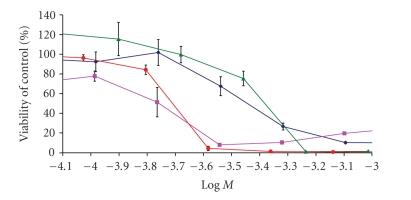
Dose-response curves for functionalized ferrocene complexes on HT-29 colon adenocarcinoma cells at 72 hours. Complex with terminal alcohol (diamonds), complex with terminal allyl (squares), ferrocene (triangles), and ferrocenium ion (circles).

Other ferrocenyl complexes, varying the lipophilic character of the carboalkoxy groups, were also evaluated for cytotoxic activity against HT-29 cells. Ferrocene complexes with either terminal alcohol or allyl groups were tested in concentrations that ranged from 13 to 1300 *μ*M at a 72-hour time interval. IC_50_ values for these complexes are also summarized in Table 2, and Figure 4 shows the cytotoxic curves for these complexes along with ferrocene and ferrocenium for comparison. The ferrocene alcohol complex shows an IC_50_ value of 370 *μ*M which is comparable to ferrocene, while (allyl) complex shows higher cytotoxic activity than ferrocene and equal to the ferrocenium at the time interval studied, with an IC_50_ value of 180 *μ*M.

**Figure 5:**
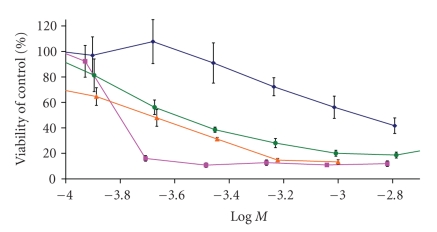
Dose-response curves for functionalized ferrocene complexes on MCF-7 breast cancer cells at 72 hours. Ferrocene(diamonds), ferrocenium(squares), bis(carboethoxy)(triangles), and terminal allyl (circles).

The initial work performed by Kopf-Maier et al. demonstrated that ferrocenium possesses in vivo anticancer activity in breast cancer [9]. Based on this precedent, we investigated our functionalized ferrocenes in MCF-7 breast cancer cell line. Surprisingly, ferrocene showed to be less cytotoxic in breast cancer than in colon cancer. With regard to the functionalized ferrocenes and similar to the results on HT-29 cell line, a pattern in the cytotoxicity as we change the functional group (pendant group) is evident. First, in the ferrocenyl with carboalkoxy groups, the incorporation of methyl ester (–CO_2_CH_3_) groups on the Cp rings decreases the cytotoxic activity of the resulting complexes. Second, see Figure 5, the increase in the lipophilic character on the carboalkoxy substituents such as in Fe(C_5_H_4_CO_2_CH_2_CH=CH_2_)_2_, where the ethyl group is substituted by an olefin, increases the cytotoxic activity. Such behavior has been reported previously by two-independent research groups, as described below [12, 25].

A wide variety of ferrocenyl derivatives have been synthesized by several research groups aimed to improve their anticancer activity as well as to understand the structure-activity relationship. Very active ferrocenyl derivatives have been synthesized containing estrogen receptor modulators as pendant groups [26]. Among them, 1,1′-bis-(4′-hydroxyphenyl)-2-ferrocenyl-but-1-ene showed strong antiproliferative activity in both hormone-dependent (MCF-7) and hormone-independent (MDA-MDA231) breast cancer cells with IC_50_ = 0.7 and 0.6 *μ*M, respectively [26]. On the other hand, ferrocenyl carbohydrate conjugates prepared by Orvig et al. showed IC_50_ values between 87–468 *μ*M on HTB-129 human breast cancer cell line [27]. Our ferrocenyl esters showed IC_50_ values similar to those of the ferrocenyl carbohydrates [27] and ferrocenium derivatives tested on MCF-7 cell line: ferroceniumcarboxylic acid tetrafluoroborate (IC_50_ = 340 *μ*M), decamethylferrocene tetrafluoroborate (IC_50_ = 37 *μ*M), 1,1′-dimethylferrocenium tetrafluoroborate (IC_50_ = 320 *μ*M), and ferrocenium boronic acid tetrafluoroborateIC_50_ = 317 *μ*M) [12]. It should be clearly noticed that, as reported previously, the increase in the lipophilic character on the ferrocene or ferrocenium complex increases the cytotoxic activity [12, 25].

For colon cancer, the cytotoxic data is more limited for a more meaningful comparison and assessment. On a recent review on the medicinal properties of ferrocenes, it was described a series of ferrocene conjugates (functionalized) anchored to polyaspartimides (water soluble carrier polymers) and evaluated on Colo and Hela colon cancer cell lines [11, 28]. The IC_50_ values determined are in the 10^−4^–10^−5^ M range, analogous to some of our most active species. From these studies, Neuse et al. found that increasing the hydrophilicity of the polyaspartimide side chains does not necessarily improve the cytotoxicity of the resulting ferrocenes, similar to our findings [28]. On the other hand, opposite to our findings, Kenny et al. studied the antiproliferative effect of a series of N-(ferrocenyl)benzoyl dipeptide esters on H1229 lung cancer cells and found that increasing the alkyl chain length of the amino acid lower the cytotoxic activity [29]. However, since the data come from different cancer cell lines, the comparison of the IC_50_ values becomes less accurate.

Finally, an important conclusion derived from this study is that minor changes in the pendant group on the Cp ring may have notable impact in the cytotoxic activity of the resulting ferrocenes. This is in good agreement with previous reports [12, 25]. Increasing the lipophilic character of the ferrocenyl esters apparently improves their biological activity, as a result of better transport of these species into the target place inside the cell [12, 13, 25]. This increased lipophilic character on the ferrocenes increases the cell uptake (improving cell membrane permeability of the ferrocene), and more ferrocene molecules could become available inside the cell to express their activity. It has been proposed by Lander et al. that not only ferrocenium cation but also ferrocene is able to express oxidative stress [15]. Ferrocene is able to generate H_2_O_2_ by autooxidation, forming ferrocenium ions. The immune-stimulatory properties of ferrocene are postulated to be mediated by redox-sensitive signaling proteins [15].

## 3. Experimental

### 3.1. General Procedure

All reactions were performed under an atmosphere of dry nitrogen using Schlenk glassware or a glovebox, unless otherwise stated. Reaction vessels were flame dried under a stream of nitrogen, and anhydrous solvents were transferred by oven-dried syringes or cannula. Tetrahydrofuran was dried and deoxygenated by distillation over K-benzophenone under nitrogen. Infrared spectra were recorded on a Brucker Vector-22 spectrometer with the samples as compressed KBr discs. The NMR spectra were obtained on a 500 MHz Bruker spectrometer. Elemental analyses were obtained from Atlantic Microlab, Inc., Ga, USA. Electrochemical characterization was carried out on a BAS CV050W voltammetric analyzer of Bioanalytical Systems, Inc. with a three-stand electrode cell. Cyclic voltammetric experiments were performed in deoxygenated CH_3_CN solution of ferrocene complexes with 1M of [NBu^n^_4_PF_6_ as supporting electrolyte and ferrocene complex concentration of 2 × 10^−3^M. The three electrodes used were platinum disk as the working electrode, Ag/AgCl as a reference electrode, and Pt wire as an auxiliary electrode. The working electrode was polished with 0.05 *μ*m alumina slurry for 1–2 minutes, and then rinsed with double-distilled and deionized water. This cleaning process is done before each CV experiment and a sweep between 0 and 2000 mV is performed on the electrolyte solution to detect any possible deposition of ferrocene on the electrode surface.

Dimethyl carbonate, diethyl carbonate, diallyl carbonate, ethylene carbonate, diethyl ether (anhydrous ≥99.7%), anhydrous FeCl_2_, [Cp_2_Fe]BF_4_, and CDCl_3_ were purchased from Sigma-Aldrich. NaCp was prepared in situ by reacting freshly distilled cyclopentadiene and NaH in THF. Silica gel was heated at about 200°C while a slow stream of dry nitrogen was passed through it.

The syntheses of sodium 1-carbomethoxycyclopentadienide (NaCpCOOCH_3_) and sodium 1-carboethoxycyclopentadienide (NaCpCOOCH_2_CH_3_) have been reported previously by our group [18].

The colon cancer cell line HT-29 and the breast adenocarcinoma cell line MCF7 were purchased from American Type Culture Collection, Va, USA, and were at 37°C and 95% Air/5% CO_2_. Growth medium for HT-29 was McCoy's 5A complete medium supplemented with 10% (v/v) fetal bovine serum and 1% (v/v) antibiotic/antimycotic. Growth medium for MCF7 was Eagle's Minimum Essential Media supplemented with 10% (v/v) fetal bovine serum, 1% (v/v) antibiotic/antimycotic, nonessential amino acids, and 0.01 mg/mL bovine insulin. MTT and Triton X-100 used for the cytotoxic assay were obtained from Sigma. All MTT manipulations were performed in a dark room.

### 3.2. Synthesis

#### 3.2.1. Synthesis of 1,1′-bis(carbomethoxy)-ferrocene (Fe(CpCOOCH_3_)_2_)

To a solution of NaCpCOOCH_3_ (0.47 g, 3.1 mmol) in THF 20 mL, was added solid FeCl_2_ (0.2 g, 1.5 mmol). The solution was stirred for 24 hours at room temperature. The solvent was removed under vacuum, and CH_2_Cl_2_ was added; the red suspension was first filtered on a Celite pad and then chromatographed on silica gel eluting with Et_2_O to give 0.45 g (95%) of orange viscous oil. The product was redissolved in chloroform/hexane (1:9) at −20°C and orange solid could be obtained.^1^HNMR (500 MHz, CDCl_3_): *δ*(ppm) 3.85(s, 6H; –OCH_3_), 4.43[t, 4 H, ^3^ *J*(H, H) = 1.5 Hz, AA′BB′; Cp], 4.85[t, 4H, ^3^ *J* (H, H) = 1.5 Hz; AA′BB′; Cp]. ^13^CNMR(125 MHz, CDCl_3_): *δ*(ppm) 51.71(–OCH_3_), 71.61(CH; Cp), 72.62(CH; Cp), 72.89(*ipso-C*; Cp), 170.82(C=O). IR (KBr, cm^−1^): 2957, 1703, 1470, 1288, 1197, 1147, 965, 780. Anal. Calcd for C_14_H_14_O_4_Fe: C, 55.67; H, 4.64. Found: C, 56.01; H, 4.74.

#### 3.2.2. Synthesis of 1,1′-bis(carboethoxy)-ferrocene (Fe(CpCOOCH_2_CH_3_)_2_

To a solution of NaCpCOOCH_2_CH_3_ (0.50 g, 3.16 mmol) in THF 20 mL, was added solid FeCl_2_ (0.2 g, 1.58 mmol). The solution was stirred for 24 hours at room temperature. The solvent was removed under vacuum, and CH_2_Cl_2_ was added; the red suspension was first filtered on a Celite pad and then chromatographed on silica gel eluting with Et_2_O to give 0.48 g (92%) of orange viscous oil. The product was resolved in chloroform/hexane (1:12) at −20°C and orange solid could be obtained. ^1^HNMR (500 MHz, CDCl_3_): *δ*(ppm) 1.38(t, 6H, ^3^ *J* (H, H) = 7.0 Hz; –OCH_2_CH_3_), 4.31(q, 4H, ^3^ *J* (H, H) = 7.0 Hz; –OCH_2_CH_3_), 4.42[t, 4H, ^3^ *J* (H, H) = 1.5 Hz, AA′BB′; Cp], 4.84[t, 4H, ^3^ *J* (H, H) = 1.5 Hz; AA′BB′; Cp]. ^13^CNMR(125 MHz, CDCl_3_): *δ*(ppm) 14.53(–OCH_2_CH_3_), 60.43(–OCH_2_CH_3_), 71.53(CH; Cp), 72.71(CH; Cp), 73.16(*ipso-C*; Cp), 170.43(C=O). IR (KBr, cm^−1^): 2985, 2936, 1708, 1479, 1457, 1379, 1282, 1144, 1030, 1017, 774. Anal. Calcd for C_16_H_18_O_4_Fe: C, 58.22; H, 5.46. Found: C, 58.19; H, 5.48.

#### 3.2.3. Synthesis of 1-(carbomethoxy)-1′-(carboethoxy)-ferrocene (Fe(CpCOOCH_3_) (CpCOOCH_2_CH_3_)

To a solution of NaCpCOOCH_3_ (0.23 g, 1.58 mmol) and NaCpCOOCH_2_CH_3_ (0.25 g, 1.58 mmol) in THF 20 mL, was added solid FeCl_2_ (0.2 g, 1.58 mmol). The solution was stirred for 24 hours at room temperature. The solvent was removed under vacuum, and CH_2_Cl_2_ was added; the red suspension was first filtered on a Celite pad and then chromatographed on silica gel eluting with Et_2_O to give 0.46 g (92%) of orange viscous oil. The product was dissolved in chloroform/hexane (1:10) at −20°C and orange solid could be obtained.^1^HNMR (500 MHz, CDCl_3_): *δ*(ppm) 1.39(t, 3H, ^3^ *J* (H, H) = 7.0 Hz; –OCH_2_CH_3_), 3.85, 3.86*(s, 3H; –OCH_3_), 4.32(q, 2H, ^3^ *J* (H, H) = 7.0 Hz; –OCH_2_CH_3_), 4.43[m, 4H; Cp], 4.85[m, 4H; Cp].^13^CNMR(125 MHz, CDCl_3_): *δ*(ppm) 14.52, 14.55*(–OCH_2_CH_3_), 51.76(–OCH_3_), 60.46, 60.48*(–OCH_2_CH_3_), 71.57, 71.61*(CH; Cp), 72.73, 72.77*(CH; Cp), 72.86, 73.21*(*ipso-C*; Cp), 170.43, 170.49*(C=O), 170.87, 170.95*(C=O). IR (KBr, cm^−1^): 2995, 2950, 1712, 1472, 1284, 1143, 1030, 774, 514. Anal. Calcd for C_15_H_16_O_4_Fe: C, 57.00; H, 5.07. Found: C, 57.19; H, 5.08.

The two complexes [Fe(C_5_H_4_CO_2_CH_2_CH=CH_2_)_2_] and [Fe{C_5_H_4_CO_2_(CH_2_)_2_OH}_2_] were prepared as described by Busetto et al. from sodium cyclopentadenide and diallyl carbonate or solid ethylene carbonate [17].

### 3.3. Cytotoxic Assay

Biological activity was determined using the MTT assay originally described by Mossman [19a] but using 10% Triton in isopropanol as a solvent for the MTT formazan crystals [19b]. HT-29 and MCF7 cells were maintained at 37°C and 95% Air/5% CO_2_ in McCoy's 5A (ATCC) complete medium, which had been supplemented with 10% (v/v) fetal bovine serum (ATCC) and 1% (v/v) antibiotic/antimycotic (Sigma). Asynchronously, growing cells were seeded at 1.5 × 10^4^ cells per well in 96-well plates containing 100 *μ*L of complete growth medium, and allowed to recover overnight. Various concentrations of the complexes (10–1300 *μ*M) dissolved in 5% DMSO/95% Medium were added to the wells (eight wells per concentration, experiments performed in quadruplicate plates). The complexes solutions were prepared first by dissolving the corresponding ferrocene in DMSO and then Medium was added to a final composition of 5% DMSO/95% Medium. In addition to the cells treated with the ferrocenes, two controls experiments were run one without any addition of solvent mixture (5% DMSO/95% Medium) and one adding 5% DMSO/95% Medium to the cells. Both control experiments behaved identical, showing that 5% of DMSO in the Medium did not render toxic to these types of cells. The cells were incubated for an additional 70 hours. After this time, MTT dissolved in complete growth medium was added to each well to a final concentration of 1.0 mg/mL and incubated for two additional hours. After this period of time, all MTTs containing medium were removed, cells were washed with cold PBS and dissolved with 200 *μ*L of a 10% (v/v) Triton X-100 solution in isopropanol. After complete dissolution of the formazan crystals, well absorbances were recorded in triplicates on a 340 ATTC Microplate Reader (SLT Lab Instruments) at 570 nm with background subtraction at 630 nm. Concentrations of compounds required to inhibit cell proliferation by 50% (IC_50_) were calculated by fitting data to a four-parameter logistic plot by means of SigmaPlot software from SPSS, Ill, USA.

### 3.4. X-Ray Crystallographic Analysis

A light orange needle crystal with 0.15 × 0.04 × 0.01 mm in size was mounted on a cryoloop with Paratone oil. Data was collected in a nitrogen gas stream at −173°C on a Bruker Smart system. Data collection was 99.6% complete to 25° in *θ*. The data was integrated using the Bruker SAINT software program. The structure was solved by direct methods and all nonhydrogen atoms were refined anisotropically by full-matrix least-squares (SHELXL-97). The crystal structure has been deposited at the Cambridge Crystallographic Data Centre and the deposition number is CCDC 705387.

## Supplementary Material

Supplementary material contains crystallography data, bonding parameters, and torsion angles for Fe(C_5_H_4_CO_2_CH_3_)_2_ and cytotoxic plot for ferrocene on HT-29 colon cancer cells.

## Figures and Tables

**Table 1:** Redox potential of ferrocenyl esters in CH_3_CN 1M CH_3_CN 1M [NBu^n^_4_]PF_6_ at a scan rate of 100 mV/s, referenced to ferrocene/ferrocenium redox couple. Ferrocene concentration of 2 × 10^−3^ M. E_1/2_ is an average of the anodic and cathodic peaks potentials.

Complex	E_1/2_ (mV)	ΔE (mV)
Fe(C_5_H_4_CO_2_CH_3_)_2_	454	75
Fe(CpCOOCH_2_CH_3_)_2_	448	74
Fe(CpCOOCH_3_)(CpCOOCH_2_CH_3_)	452	78
Fe(C_5_H_4_CO_2_CH_2_CH=CH_2_)_2_	453	85
Fe(C_5_H_4_CO_2_CH_2_CH_2_OH)_2_	456	89

**Table 2:** Cytotoxicities of ferrocenes studied on HT-29 colon cancer and MCF-7 breast cancer cell lines at 72 hours, as determined by MTT assay. IC values are the average of four independent measurements with their standard deviations ().

	HT-29	MCF-7
Complex	IC_50_ (*μ*M)	IC_50_ (*μ*M)
FeCp_2_	360(30)	1500(100)
Fe(Cp–COOEt)_2_	370(10)	250(20)
Fe(Cp–COOMe)(Cp–COOEt)	500(20)	320(30)
Fe(Cp–COOMe)_2_	720(50)	520(20)
Fe(CpCOOCH_2_CH_2_OH)_2_	370(20)	340(30)
Fe(CpCOCH_2_CHCH_2_)_2_	180(10)	190(30)
Fe[Cp_2_]BF_4_	180(10)	150(05)
