# Supplementary material for: Synthesis, Structure, Electrochemistry, and Cytotoxic Properties of Ferrocenyl Ester Derivatives
Source: Met Based Drugs. 2009 Mar 24;2009:420784. doi: 10.1155/2009/420784 (PMC2659881; doi:10.1155/2009/420784)
Supplement: Supplementary file 1 — Supplementary material contains crystallography data, bonding parameters, and torsion angles for Fe(C5H4CO2CH3)2 and cytotoxic plot for ferrocene on HT-29 colon cancer cells. [file 420784.f1.rtf]

Supplementary material contains crystallography data, bonding parameters and torsion angles for Fe(C5H4CO2CH3)2 and cytotoxic plot for ferrocene on HT-29 colon cancer cells.


Table 1.  Crystal data and structure refinement for Fe(CpCOOCH3)2.

Empirical formula 	C14 H14 Fe O4
Formula weight 	302.10
Temperature 	173(2) K
Wavelength 	0.71073 Å
Crystal system 	Monoclinic
Space group 	C2/c
Unit cell dimensions	a = 32.340(5) Å	a= 90°.
	b = 5.9084(9) Å	b= 103.185(2)°.
	c = 13.2240(19) Å	g = 90°.
Volume	2460.2(6) Å3
Z	8
Density (calculated)	1.631 Mg/m3
Absorption coefficient	1.232 mm-1
F(000)	1248
Crystal size	0.15 x 0.04 x 0.01 mm3
Theta range for data collection	1.29 to 28.28°.
Index ranges	-41<=h<=28, -7<=k<=7, -14<=l<=17
Reflections collected	6828
Independent reflections	2782 [R(int) = 0.0443]
Completeness to theta = 25.00°	99.6 % 
Absorption correction	None
Max. and min. transmission	0.9878 and 0.8367
Refinement method	Full-matrix least-squares on F2
Data / restraints / parameters	2782 / 0 / 229
Goodness-of-fit on F2	0.972
Final R indices [I>2sigma(I)]	R1 = 0.0397, wR2 = 0.0713
R indices (all data)	R1 = 0.0774, wR2 = 0.0832
Extinction coefficient	0.00000(8)
Largest diff. peak and hole	0.619 and -0.357 e.Å-3

Table 2.  Atomic coordinates ( x 104) and equivalent isotropic displacement parameters (Å2x 103)
for Fe(CpCOOCH3)2.  U(eq) is defined as one third of the trace of the orthogonalized Uij tensor.
________________________________________________________________________________
	x	y	z	U(eq)
________________________________________________________________________________
Fe(1)	1262(1)	2481(1)	8490(1)	13(1)
C(7)	1029(1)	3899(5)	7073(2)	14(1)
C(11)	585(1)	4636(5)	6669(2)	17(1)
C(8)	1379(1)	5200(6)	7642(2)	17(1)
C(6)	1176(1)	1668(6)	6952(2)	18(1)
C(1)	1120(1)	3735(6)	9806(2)	18(1)
C(13)	1928(1)	3018(5)	10579(2)	16(1)
C(14)	2395(1)	6062(6)	11147(3)	21(1)
C(4)	1378(1)	120(5)	9661(2)	17(1)
C(5)	1492(1)	2339(6)	10052(2)	16(1)
C(3)	936(1)	145(6)	9167(2)	18(1)
C(10)	1616(1)	1596(6)	7433(2)	20(1)
C(9)	1738(1)	3762(6)	7864(2)	19(1)
C(12)	121(1)	7759(7)	6298(3)	24(1)
C(2)	780(1)	2370(6)	9269(2)	19(1)
O(1)	299(1)	3399(4)	6242(2)	24(1)
O(4)	1977(1)	5267(3)	10607(2)	18(1)
O(3)	2210(1)	1702(4)	10943(2)	22(1)
O(2)	538(1)	6871(3)	6790(2)	20(1)
________________________________________________________________________________
Table 3.   Bond lengths [Å] and angles [°] for Fe(CpCOOCH3)2.
_____________________________________________________
Fe(1)-C(5) 	2.031(3)
Fe(1)-C(7) 	2.034(3)
Fe(1)-C(1) 	2.038(3)
Fe(1)-C(8) 	2.043(3)
Fe(1)-C(6) 	2.047(3)
Fe(1)-C(9) 	2.051(3)
Fe(1)-C(4) 	2.054(3)
Fe(1)-C(2) 	2.058(3)
Fe(1)-C(3) 	2.062(3)
Fe(1)-C(10) 	2.064(3)
C(7)-C(6) 	1.423(4)
C(7)-C(8) 	1.431(4)
C(7)-C(11) 	1.477(4)
C(5)-C(13)	1.477(4)
C(11)-O(1) 	1.215(3)
C(11)-O(2) 	1.343(4)
C(8)-C(9) 	1.415(4)
C(8)-H(8) 	0.92(3)
C(6)-C(10) 	1.420(4)
C(6)-H(6) 	0.87(3)
C(1)-C(2) 	1.417(4)
C(1)-C(5) 	1.434(4)
C(1)-H(1) 	0.92(3)
C(13)-O(3) 	1.212(3)
C(13)-O(4) 	1.338(3)
C(13)-C(5) 	1.477(4)
C(14)-O(4) 	1.456(3)
C(14)-H(14) 	0.96(3)
C(14)-H(16) 	0.95(3)
C(14)-H(15) 	1.00(3)
C(4)-C(5) 	1.427(4)
C(4)-C(3) 	1.432(4)
C(4)-H(4) 	0.94(3)
C(3)-C(2) 	1.425(5)
C(3)-H(3) 	0.92(3)
C(10)-C(9) 	1.420(5)
C(10)-H(10) 	0.96(3)
C(9)-H(9) 	0.96(3)
C(12)-O(2) 	1.454(4)
C(12)-H(11) 	0.96(3)
C(12)-H(12) 	0.96(3)
C(12)-H(13) 	0.95(3)
C(2)-H(2) 	0.91(3)

C(5)-Fe(1)-C(7)	157.89(13)
C(5)-Fe(1)-C(1)	41.27(12)
C(7)-Fe(1)-C(1)	121.85(12)
C(5)-Fe(1)-C(8)	121.34(13)
C(7)-Fe(1)-C(8)	41.10(11)
C(1)-Fe(1)-C(8)	106.76(13)
C(5)-Fe(1)-C(6)	159.48(12)
C(7)-Fe(1)-C(6)	40.82(12)
C(1)-Fe(1)-C(6)	158.08(12)
C(8)-Fe(1)-C(6)	68.86(13)
C(5)-Fe(1)-C(9)	106.95(12)
C(7)-Fe(1)-C(9)	68.35(12)
C(1)-Fe(1)-C(9)	123.15(13)
C(8)-Fe(1)-C(9)	40.45(12)
C(6)-Fe(1)-C(9)	68.29(13)
C(5)-Fe(1)-C(4)	40.87(12)
C(7)-Fe(1)-C(4)	160.01(12)
C(1)-Fe(1)-C(4)	68.97(13)
C(8)-Fe(1)-C(4)	157.40(12)
C(6)-Fe(1)-C(4)	123.38(13)
C(9)-Fe(1)-C(4)	122.04(12)
C(5)-Fe(1)-C(2)	68.47(11)
C(7)-Fe(1)-C(2)	108.03(12)
C(1)-Fe(1)-C(2)	40.48(12)
C(8)-Fe(1)-C(2)	123.74(13)
C(6)-Fe(1)-C(2)	122.90(12)
C(9)-Fe(1)-C(2)	159.75(14)
C(4)-Fe(1)-C(2)	68.27(12)
C(5)-Fe(1)-C(3)	68.66(12)
C(7)-Fe(1)-C(3)	123.78(12)
C(1)-Fe(1)-C(3)	68.59(13)
C(8)-Fe(1)-C(3)	160.25(12)
C(6)-Fe(1)-C(3)	107.89(13)
C(9)-Fe(1)-C(3)	158.12(13)
C(4)-Fe(1)-C(3)	40.70(11)
C(2)-Fe(1)-C(3)	40.48(13)
C(5)-Fe(1)-C(10)	123.20(12)
C(7)-Fe(1)-C(10)	68.12(12)
C(1)-Fe(1)-C(10)	159.70(12)
C(8)-Fe(1)-C(10)	68.15(13)
C(6)-Fe(1)-C(10)	40.41(12)
C(9)-Fe(1)-C(10)	40.37(13)
C(4)-Fe(1)-C(10)	107.74(13)
C(2)-Fe(1)-C(10)	158.60(13)
C(3)-Fe(1)-C(10)	122.82(13)
C(6)-C(7)-C(8)	108.2(3)
C(6)-C(7)-C(11)	123.7(3)
C(8)-C(7)-C(11)	128.1(3)
C(6)-C(7)-Fe(1)	70.06(18)
C(8)-C(7)-Fe(1)	69.77(16)
C(11)-C(7)-Fe(1)	125.51(19)
O(1)-C(11)-O(2)	123.4(3)
O(1)-C(11)-C(7)	124.5(3)
O(2)-C(11)-C(7)	112.0(3)
C(9)-C(8)-C(7)	107.5(3)
C(9)-C(8)-Fe(1)	70.09(18)
C(7)-C(8)-Fe(1)	69.13(17)
C(9)-C(8)-H(8)	128.3(18)
C(7)-C(8)-H(8)	124.3(17)
Fe(1)-C(8)-H(8)	125.3(18)
C(10)-C(6)-C(7)	107.7(3)
C(10)-C(6)-Fe(1)	70.45(18)
C(7)-C(6)-Fe(1)	69.12(17)
C(10)-C(6)-H(6)	123(2)
C(7)-C(6)-H(6)	129(2)
Fe(1)-C(6)-H(6)	127(2)
C(2)-C(1)-C(5)	107.6(3)
C(2)-C(1)-Fe(1)	70.49(17)
C(5)-C(1)-Fe(1)	69.08(16)
C(2)-C(1)-H(1)	126.5(19)
C(5)-C(1)-H(1)	126.0(19)
Fe(1)-C(1)-H(1)	125.5(19)
O(3)-C(13)-O(4)	123.6(3)
O(3)-C(13)-C(5)	124.3(3)
O(4)-C(13)-C(5)	112.2(3)
O(4)-C(14)-H(14)	110.4(17)
O(4)-C(14)-H(16)	107.6(19)
H(14)-C(14)-H(16)	112(3)
O(4)-C(14)-H(15)	104.7(17)
H(14)-C(14)-H(15)	112(3)
H(16)-C(14)-H(15)	109(3)
C(5)-C(4)-C(3)	107.7(3)
C(5)-C(4)-Fe(1)	68.68(17)
C(3)-C(4)-Fe(1)	69.93(17)
C(5)-C(4)-H(4)	128.9(18)
C(3)-C(4)-H(4)	123.4(18)
Fe(1)-C(4)-H(4)	126.5(18)
C(4)-C(5)-C(1)	108.2(3)
C(4)-C(5)-C(13)	124.1(3)
C(1)-C(5)-C(13)	127.7(3)
C(4)-C(5)-Fe(1)	70.45(16)
C(1)-C(5)-Fe(1)	69.65(16)
C(13)-C(5)-Fe(1)	123.85(19)
C(2)-C(3)-C(4)	107.7(3)
C(2)-C(3)-Fe(1)	69.60(17)
C(4)-C(3)-Fe(1)	69.36(17)
C(2)-C(3)-H(3)	128.0(18)
C(4)-C(3)-H(3)	124.2(18)
Fe(1)-C(3)-H(3)	124.3(18)
C(6)-C(10)-C(9)	108.2(3)
C(6)-C(10)-Fe(1)	69.14(17)
C(9)-C(10)-Fe(1)	69.32(17)
C(6)-C(10)-H(10)	123.8(18)
C(9)-C(10)-H(10)	128.0(17)
Fe(1)-C(10)-H(10)	126.5(17)
C(8)-C(9)-C(10)	108.5(3)
C(8)-C(9)-Fe(1)	69.46(17)
C(10)-C(9)-Fe(1)	70.31(18)
C(8)-C(9)-H(9)	126.0(19)
C(10)-C(9)-H(9)	125.3(19)
Fe(1)-C(9)-H(9)	121.6(17)
O(2)-C(12)-H(11)	108.5(17)
O(2)-C(12)-H(12)	107.7(18)
H(11)-C(12)-H(12)	111(3)
O(2)-C(12)-H(13)	106.7(19)
H(11)-C(12)-H(13)	109(3)
H(12)-C(12)-H(13)	114(3)
C(1)-C(2)-C(3)	108.8(3)
C(1)-C(2)-Fe(1)	69.03(16)
C(3)-C(2)-Fe(1)	69.92(16)
C(1)-C(2)-H(2)	127.9(19)
C(3)-C(2)-H(2)	123.4(19)
Fe(1)-C(2)-H(2)	125.7(17)
C(13)-O(4)-C(14)	115.1(2)
C(11)-O(2)-C(12)	114.9(2)
_____________________________________________________________
Symmetry transformations used to generate equivalent atoms: 
 

Table 4.   Anisotropic displacement parameters (Å2x 103)for Fe(CpCOOCH3)2.  The anisotropic
displacement factor exponent takes the form: -2p2[ h2a*2U11 + ... + 2 h k a* b* U12 ]
______________________________________________________________________________
	U11	U22	U33	U23	U13	U12
______________________________________________________________________________
Fe(1)	13(1) 	16(1)	12(1) 	0(1)	3(1) 	0(1)
C(7)	18(1) 	14(2)	11(1) 	1(1)	3(1) 	-1(1)
C(11)	22(2) 	20(2)	11(2) 	4(1)	5(1) 	0(1)
C(8)	20(2) 	17(2)	15(2) 	1(1)	7(1) 	-3(1)
C(6)	22(2) 	19(2)	12(2) 	-1(1)	5(1) 	-1(1)
C(1)	21(2) 	20(2)	15(2) 	-1(1)	8(1) 	1(1)
C(13)	20(2) 	19(2)	10(1) 	0(1)	5(1) 	-1(1)
C(14)	16(2) 	21(2)	24(2) 	0(2)	3(1) 	-2(1)
C(4)	20(2) 	18(2)	14(2) 	6(1)	6(1) 	2(1)
C(5)	16(1) 	18(2)	12(1) 	3(2)	3(1) 	0(1)
C(3)	19(2) 	20(2)	16(2) 	2(1)	4(1) 	-7(1)
C(10)	21(2) 	26(2)	14(2) 	3(1)	8(1) 	5(1)
C(9)	16(2) 	26(2)	14(2) 	1(1)	6(1) 	-2(1)
C(12)	22(2) 	25(2)	24(2) 	3(2)	3(1) 	6(2)
C(2)	14(1) 	28(2)	16(1) 	5(2)	6(1) 	1(2)
O(1)	22(1) 	20(1)	28(1) 	1(1)	-4(1) 	-1(1)
O(4)	16(1) 	16(1)	19(1) 	-2(1)	-1(1) 	0(1)
O(3)	16(1) 	20(1)	28(1) 	2(1)	0(1) 	2(1)
O(2)	17(1) 	18(1)	23(1) 	-1(1)	1(1) 	2(1)
______________________________________________________________________________
Table 5.   Hydrogen coordinates ( x 104) and isotropic displacement parameters (Å2x 103)
for Fe(CpCOOCH3)2.
________________________________________________________________________________
	x 	y 	z 	U(eq)
________________________________________________________________________________

H(3)	790(9)	-1070(50)	8820(20)	16(8)
H(2)	506(9)	2790(50)	9010(20)	14(7)
H(4)	1551(9)	-1180(50)	9700(20)	19(8)
H(1)	1106(9)	5240(60)	9970(20)	22(9)
H(6)	1036(9)	510(60)	6640(20)	27(10)
H(8)	1360(8)	6700(50)	7830(20)	10(8)
H(10)	1789(9)	270(50)	7460(20)	18(8)
H(14)	2609(9)	5480(50)	10820(20)	18(8)
H(16)	2439(9)	5600(60)	11850(30)	33(10)
H(15)	2375(9)	7740(60)	11110(20)	24(9)
H(11)	-88(9)	6980(50)	6580(20)	18(8)
H(12)	77(9)	7490(50)	5570(20)	23(8)
H(13)	121(9)	9310(60)	6480(20)	29(10)
H(9)	2012(9)	4140(50)	8290(20)	20(8)
________________________________________________________________________________
Table 6.  Torsion angles [°] for Fe(CpCOOCH3)2.
________________________________________________________________
C(5)-Fe(1)-C(7)-C(6)	163.2(3)
C(1)-Fe(1)-C(7)-C(6)	-162.11(17)
C(8)-Fe(1)-C(7)-C(6)	119.2(2)
C(9)-Fe(1)-C(7)-C(6)	81.40(18)
C(4)-Fe(1)-C(7)-C(6)	-44.0(4)
C(2)-Fe(1)-C(7)-C(6)	-119.80(18)
C(3)-Fe(1)-C(7)-C(6)	-77.9(2)
C(10)-Fe(1)-C(7)-C(6)	37.79(17)
C(5)-Fe(1)-C(7)-C(8)	44.0(4)
C(1)-Fe(1)-C(7)-C(8)	78.7(2)
C(6)-Fe(1)-C(7)-C(8)	-119.2(2)
C(9)-Fe(1)-C(7)-C(8)	-37.77(18)
C(4)-Fe(1)-C(7)-C(8)	-163.1(3)
C(2)-Fe(1)-C(7)-C(8)	121.02(19)
C(3)-Fe(1)-C(7)-C(8)	162.89(18)
C(10)-Fe(1)-C(7)-C(8)	-81.39(19)
C(5)-Fe(1)-C(7)-C(11)	-79.1(4)
C(1)-Fe(1)-C(7)-C(11)	-44.3(3)
C(8)-Fe(1)-C(7)-C(11)	-123.1(3)
C(6)-Fe(1)-C(7)-C(11)	117.8(3)
C(9)-Fe(1)-C(7)-C(11)	-160.8(3)
C(4)-Fe(1)-C(7)-C(11)	73.8(4)
C(2)-Fe(1)-C(7)-C(11)	-2.0(3)
C(3)-Fe(1)-C(7)-C(11)	39.8(3)
C(10)-Fe(1)-C(7)-C(11)	155.5(3)
C(6)-C(7)-C(11)-O(1)	3.9(5)
C(8)-C(7)-C(11)-O(1)	-175.6(3)
Fe(1)-C(7)-C(11)-O(1)	-84.5(4)
C(6)-C(7)-C(11)-O(2)	-173.5(3)
C(8)-C(7)-C(11)-O(2)	6.9(4)
Fe(1)-C(7)-C(11)-O(2)	98.1(3)
C(6)-C(7)-C(8)-C(9)	0.1(3)
C(11)-C(7)-C(8)-C(9)	179.7(3)
Fe(1)-C(7)-C(8)-C(9)	59.9(2)
C(6)-C(7)-C(8)-Fe(1)	-59.8(2)
C(11)-C(7)-C(8)-Fe(1)	119.8(3)
C(5)-Fe(1)-C(8)-C(9)	79.2(2)
C(7)-Fe(1)-C(8)-C(9)	-118.7(3)
C(1)-Fe(1)-C(8)-C(9)	121.80(19)
C(6)-Fe(1)-C(8)-C(9)	-80.9(2)
C(4)-Fe(1)-C(8)-C(9)	46.4(4)
C(2)-Fe(1)-C(8)-C(9)	162.85(18)
C(3)-Fe(1)-C(8)-C(9)	-165.0(3)
C(10)-Fe(1)-C(8)-C(9)	-37.35(18)
C(5)-Fe(1)-C(8)-C(7)	-162.18(16)
C(1)-Fe(1)-C(8)-C(7)	-119.54(18)
C(6)-Fe(1)-C(8)-C(7)	37.73(16)
C(9)-Fe(1)-C(8)-C(7)	118.7(3)
C(4)-Fe(1)-C(8)-C(7)	165.0(3)
C(2)-Fe(1)-C(8)-C(7)	-78.5(2)
C(3)-Fe(1)-C(8)-C(7)	-46.4(4)
C(10)-Fe(1)-C(8)-C(7)	81.30(19)
C(8)-C(7)-C(6)-C(10)	-0.6(3)
C(11)-C(7)-C(6)-C(10)	179.8(3)
Fe(1)-C(7)-C(6)-C(10)	-60.2(2)
C(8)-C(7)-C(6)-Fe(1)	59.6(2)
C(11)-C(7)-C(6)-Fe(1)	-120.0(3)
C(5)-Fe(1)-C(6)-C(10)	-43.2(4)
C(7)-Fe(1)-C(6)-C(10)	118.7(3)
C(1)-Fe(1)-C(6)-C(10)	163.1(3)
C(8)-Fe(1)-C(6)-C(10)	80.7(2)
C(9)-Fe(1)-C(6)-C(10)	37.12(19)
C(4)-Fe(1)-C(6)-C(10)	-77.8(2)
C(2)-Fe(1)-C(6)-C(10)	-162.0(2)
C(3)-Fe(1)-C(6)-C(10)	-120.0(2)
C(5)-Fe(1)-C(6)-C(7)	-161.9(3)
C(1)-Fe(1)-C(6)-C(7)	44.4(4)
C(8)-Fe(1)-C(6)-C(7)	-37.98(16)
C(9)-Fe(1)-C(6)-C(7)	-81.58(18)
C(4)-Fe(1)-C(6)-C(7)	163.49(16)
C(2)-Fe(1)-C(6)-C(7)	79.3(2)
C(3)-Fe(1)-C(6)-C(7)	121.34(17)
C(10)-Fe(1)-C(6)-C(7)	-118.7(3)
C(5)-Fe(1)-C(1)-C(2)	-118.6(3)
C(7)-Fe(1)-C(1)-C(2)	80.4(2)
C(8)-Fe(1)-C(1)-C(2)	122.7(2)
C(6)-Fe(1)-C(1)-C(2)	47.8(4)
C(9)-Fe(1)-C(1)-C(2)	163.92(19)
C(4)-Fe(1)-C(1)-C(2)	-80.8(2)
C(3)-Fe(1)-C(1)-C(2)	-36.95(19)
C(10)-Fe(1)-C(1)-C(2)	-165.1(3)
C(7)-Fe(1)-C(1)-C(5)	-161.03(17)
C(8)-Fe(1)-C(1)-C(5)	-118.71(19)
C(6)-Fe(1)-C(1)-C(5)	166.4(3)
C(9)-Fe(1)-C(1)-C(5)	-77.5(2)
C(4)-Fe(1)-C(1)-C(5)	37.81(18)
C(2)-Fe(1)-C(1)-C(5)	118.6(3)
C(3)-Fe(1)-C(1)-C(5)	81.6(2)
C(10)-Fe(1)-C(1)-C(5)	-46.5(5)
C(7)-Fe(1)-C(4)-C(5)	-164.8(3)
C(1)-Fe(1)-C(4)-C(5)	-38.16(17)
C(8)-Fe(1)-C(4)-C(5)	45.0(4)
C(6)-Fe(1)-C(4)-C(5)	162.28(16)
C(9)-Fe(1)-C(4)-C(5)	78.6(2)
C(2)-Fe(1)-C(4)-C(5)	-81.77(18)
C(3)-Fe(1)-C(4)-C(5)	-119.4(2)
C(10)-Fe(1)-C(4)-C(5)	120.58(17)
C(5)-Fe(1)-C(4)-C(3)	119.4(3)
C(7)-Fe(1)-C(4)-C(3)	-45.4(4)
C(1)-Fe(1)-C(4)-C(3)	81.2(2)
C(8)-Fe(1)-C(4)-C(3)	164.3(3)
C(6)-Fe(1)-C(4)-C(3)	-78.3(2)
C(9)-Fe(1)-C(4)-C(3)	-162.02(19)
C(2)-Fe(1)-C(4)-C(3)	37.60(19)
C(10)-Fe(1)-C(4)-C(3)	-120.04(19)
C(3)-C(4)-C(5)-C(1)	0.4(3)
Fe(1)-C(4)-C(5)-C(1)	59.7(2)
C(3)-C(4)-C(5)-C(13)	-177.5(3)
Fe(1)-C(4)-C(5)-C(13)	-118.3(3)
C(3)-C(4)-C(5)-Fe(1)	-59.2(2)
C(2)-C(1)-C(5)-C(4)	0.1(3)
Fe(1)-C(1)-C(5)-C(4)	-60.2(2)
C(2)-C(1)-C(5)-C(13)	177.9(3)
Fe(1)-C(1)-C(5)-C(13)	117.7(3)
C(2)-C(1)-C(5)-Fe(1)	60.3(2)
O(3)-C(13)-C(5)-C(4)	-17.4(5)
O(4)-C(13)-C(5)-C(4)	162.3(3)
O(3)-C(13)-C(5)-C(1)	165.1(3)
O(4)-C(13)-C(5)-C(1)	-15.2(4)
O(3)-C(13)-C(5)-Fe(1)	-105.6(3)
O(4)-C(13)-C(5)-Fe(1)	74.2(3)
C(7)-Fe(1)-C(5)-C(4)	166.2(3)
C(1)-Fe(1)-C(5)-C(4)	119.0(2)
C(8)-Fe(1)-C(5)-C(4)	-161.46(17)
C(6)-Fe(1)-C(5)-C(4)	-46.5(4)
C(9)-Fe(1)-C(5)-C(4)	-119.69(18)
C(2)-Fe(1)-C(5)-C(4)	81.23(19)
C(3)-Fe(1)-C(5)-C(4)	37.60(17)
C(10)-Fe(1)-C(5)-C(4)	-78.5(2)
C(7)-Fe(1)-C(5)-C(1)	47.2(4)
C(8)-Fe(1)-C(5)-C(1)	79.5(2)
C(6)-Fe(1)-C(5)-C(1)	-165.5(3)
C(9)-Fe(1)-C(5)-C(1)	121.3(2)
C(4)-Fe(1)-C(5)-C(1)	-119.0(2)
C(2)-Fe(1)-C(5)-C(1)	-37.80(19)
C(3)-Fe(1)-C(5)-C(1)	-81.4(2)
C(10)-Fe(1)-C(5)-C(1)	162.48(19)
C(7)-Fe(1)-C(5)-C(13)	-75.3(4)
C(1)-Fe(1)-C(5)-C(13)	-122.4(4)
C(8)-Fe(1)-C(5)-C(13)	-42.9(3)
C(6)-Fe(1)-C(5)-C(13)	72.0(5)
C(9)-Fe(1)-C(5)-C(13)	-1.2(3)
C(4)-Fe(1)-C(5)-C(13)	118.5(3)
C(2)-Fe(1)-C(5)-C(13)	-160.2(3)
C(3)-Fe(1)-C(5)-C(13)	156.1(3)
C(10)-Fe(1)-C(5)-C(13)	40.0(3)
C(5)-C(4)-C(3)-C(2)	-0.8(3)
Fe(1)-C(4)-C(3)-C(2)	-59.2(2)
C(5)-C(4)-C(3)-Fe(1)	58.5(2)
C(5)-Fe(1)-C(3)-C(2)	81.42(19)
C(7)-Fe(1)-C(3)-C(2)	-77.9(2)
C(1)-Fe(1)-C(3)-C(2)	36.95(17)
C(8)-Fe(1)-C(3)-C(2)	-42.9(5)
C(6)-Fe(1)-C(3)-C(2)	-120.06(18)
C(9)-Fe(1)-C(3)-C(2)	163.8(3)
C(4)-Fe(1)-C(3)-C(2)	119.2(3)
C(10)-Fe(1)-C(3)-C(2)	-161.99(17)
C(5)-Fe(1)-C(3)-C(4)	-37.75(18)
C(7)-Fe(1)-C(3)-C(4)	162.96(17)
C(1)-Fe(1)-C(3)-C(4)	-82.2(2)
C(8)-Fe(1)-C(3)-C(4)	-162.1(3)
C(6)-Fe(1)-C(3)-C(4)	120.76(19)
C(9)-Fe(1)-C(3)-C(4)	44.6(4)
C(2)-Fe(1)-C(3)-C(4)	-119.2(3)
C(10)-Fe(1)-C(3)-C(4)	78.8(2)
C(7)-C(6)-C(10)-C(9)	0.9(3)
Fe(1)-C(6)-C(10)-C(9)	-58.5(2)
C(7)-C(6)-C(10)-Fe(1)	59.3(2)
C(5)-Fe(1)-C(10)-C(6)	163.35(19)
C(7)-Fe(1)-C(10)-C(6)	-38.16(18)
C(1)-Fe(1)-C(10)-C(6)	-161.7(3)
C(8)-Fe(1)-C(10)-C(6)	-82.6(2)
C(9)-Fe(1)-C(10)-C(6)	-120.0(3)
C(4)-Fe(1)-C(10)-C(6)	121.0(2)
C(2)-Fe(1)-C(10)-C(6)	45.5(4)
C(3)-Fe(1)-C(10)-C(6)	78.8(2)
C(5)-Fe(1)-C(10)-C(9)	-76.6(2)
C(7)-Fe(1)-C(10)-C(9)	81.88(19)
C(1)-Fe(1)-C(10)-C(9)	-41.7(4)
C(8)-Fe(1)-C(10)-C(9)	37.43(17)
C(6)-Fe(1)-C(10)-C(9)	120.0(3)
C(4)-Fe(1)-C(10)-C(9)	-118.93(18)
C(2)-Fe(1)-C(10)-C(9)	165.5(3)
C(3)-Fe(1)-C(10)-C(9)	-161.13(17)
C(7)-C(8)-C(9)-C(10)	0.4(3)
Fe(1)-C(8)-C(9)-C(10)	59.7(2)
C(7)-C(8)-C(9)-Fe(1)	-59.3(2)
C(6)-C(10)-C(9)-C(8)	-0.8(3)
Fe(1)-C(10)-C(9)-C(8)	-59.2(2)
C(6)-C(10)-C(9)-Fe(1)	58.4(2)
C(5)-Fe(1)-C(9)-C(8)	-118.72(19)
C(7)-Fe(1)-C(9)-C(8)	38.36(18)
C(1)-Fe(1)-C(9)-C(8)	-76.4(2)
C(6)-Fe(1)-C(9)-C(8)	82.5(2)
C(4)-Fe(1)-C(9)-C(8)	-160.84(18)
C(2)-Fe(1)-C(9)-C(8)	-45.1(4)
C(3)-Fe(1)-C(9)-C(8)	166.5(3)
C(10)-Fe(1)-C(9)-C(8)	119.6(3)
C(5)-Fe(1)-C(9)-C(10)	121.67(18)
C(7)-Fe(1)-C(9)-C(10)	-81.24(19)
C(1)-Fe(1)-C(9)-C(10)	164.00(17)
C(8)-Fe(1)-C(9)-C(10)	-119.6(3)
C(6)-Fe(1)-C(9)-C(10)	-37.16(18)
C(4)-Fe(1)-C(9)-C(10)	79.6(2)
C(2)-Fe(1)-C(9)-C(10)	-164.7(3)
C(3)-Fe(1)-C(9)-C(10)	46.8(4)
C(5)-C(1)-C(2)-C(3)	-0.6(3)
Fe(1)-C(1)-C(2)-C(3)	58.8(2)
C(5)-C(1)-C(2)-Fe(1)	-59.4(2)
C(4)-C(3)-C(2)-C(1)	0.9(3)
Fe(1)-C(3)-C(2)-C(1)	-58.2(2)
C(4)-C(3)-C(2)-Fe(1)	59.1(2)
C(5)-Fe(1)-C(2)-C(1)	38.52(19)
C(7)-Fe(1)-C(2)-C(1)	-118.3(2)
C(8)-Fe(1)-C(2)-C(1)	-75.6(2)
C(6)-Fe(1)-C(2)-C(1)	-160.76(19)
C(9)-Fe(1)-C(2)-C(1)	-42.1(4)
C(4)-Fe(1)-C(2)-C(1)	82.6(2)
C(3)-Fe(1)-C(2)-C(1)	120.5(3)
C(10)-Fe(1)-C(2)-C(1)	165.9(3)
C(5)-Fe(1)-C(2)-C(3)	-81.9(2)
C(7)-Fe(1)-C(2)-C(3)	121.29(18)
C(1)-Fe(1)-C(2)-C(3)	-120.5(3)
C(8)-Fe(1)-C(2)-C(3)	163.93(17)
C(6)-Fe(1)-C(2)-C(3)	78.8(2)
C(9)-Fe(1)-C(2)-C(3)	-162.5(3)
C(4)-Fe(1)-C(2)-C(3)	-37.80(18)
C(10)-Fe(1)-C(2)-C(3)	45.4(4)
O(3)-C(13)-O(4)-C(14)	-2.2(4)
C(5)-C(13)-O(4)-C(14)	178.1(2)
O(1)-C(11)-O(2)-C(12)	-4.6(4)
C(7)-C(11)-O(2)-C(12)	172.9(2)
________________________________________________________________
Symmetry transformations used to generate equivalent atoms: 


Supplemenatry Material IC50


Figure 1S. Cytotoxic activity of ferrocene against HT29 colon adenocarcinoma cells at time intervals of 72 (diamonds), 96 (squares) and 120hours (triangles). Error bars omitted for clarity.
